# The Effect of Diet on Colony Recognition and Cuticular Hydrocarbon Profiles of the Invasive Argentine Ant, *Linepithema humile*

**DOI:** 10.3390/insects13040335

**Published:** 2022-03-29

**Authors:** Ellen van Wilgenburg, Mario Mariotta, Neil Durie Tsutsui

**Affiliations:** 1 Department of Natural Sciences, Fordham University, New York, NY 10023, USA; evanwilgenburg@fordham.edu; 2 Independent Researcher, Los Angeles, CA 90012, USA; Mario.mariottaiv@gmail.com; 3 Department of Environmental Science, Policy and Management, 130 Mulford Hall #3114, University of California-Berkeley, Berkeley, CA 94720-3114, USA

**Keywords:** aggression, colony recognition, cuticular hydrocarbon, invasive species, nestmate recognition, pheromone, social insect, sociality

## Abstract

**Simple Summary:**

The membership of social insect colonies is defined by chemical pheromones on the bodies of colony members. In nearly all ant species that have been studied, these pheromones have been shown to be genetically based. In some cases, however, environmentally derived odors have been implicated as colony recognition cues. The widely introduced and invasive Argentine ant is well known for forming massive “supercolonies” in its introduced ranges. Previous studies have implicated both genetic and diet-derived chemicals in the colony recognition systems of introduced populations. Here, we perform feeding experiments, in both realistic field settings and the lab, and show that dietary changes do not cause behavioral changes in the field, as well as under most laboratory conditions. However, one exception was found, in which reduced aggression was recorded in one of the laboratory feeding treatments (with crickets as the dietary item), but, nevertheless, not of sufficient magnitude to explain the unusual colony structure of introduced Argentine ants. We conclude that dietary shifts during introduction to new ranges do not account for the origin of Argentine ant supercolonies.

**Abstract:**

Ants are some of the most abundant and ecologically successful terrestrial organisms, and invasive ants rank among the most damaging invasive species. The Argentine ant is a particularly well-studied invader, in part, because of the extreme social structure, known as *unicoloniality*, that occurs in introduced populations. Unicoloniality is characterized by the formation of geographically vast supercolonies, within which territorial behavior and intraspecific aggression are absent. Although there is considerable evidence supporting a genetic basis for the odor cues involved in colony recognition, some studies have suggested that diet may also influence colony recognition cues and, thus, colony structure. Here, we test the role for insect-derived recognition cues by performing a diet supplementation experiment in a natural field setting, and a more extreme dietary manipulation experiment in the lab. After one month, in both the field and the lab, we found that aggressive supercolonies remained aggressive toward each other and non-aggressive nests (from the same supercolony) remained non-aggressive, regardless of dietary treatment. In one lab treatment, we did observe a significant decrease in the level of aggression between different supercolonies that were fed the same diet, but aggression was still frequent. We did not see any evidence for cuticular hydrocarbon odor cues being transferred from prey to ants in any of the field treatments. In the more extreme lab treatment, however, several cuticular hydrocarbons were acquired from both roach and cricket insect prey (but not *Drosophila*). Based on these data, we conclude that dietary changes are unlikely to underlie changes in behavior or colony structure in Argentine ants in real-world settings. However, these results indicate that caution is warranted when interpreting the behaviors of animals that have been reared on diets that are substantially different from natural populations.

## 1. Introduction

In response to human-induced modifications to the environment, global biodiversity is changing at an unprecedented rate, and biological invasions are recognized as one of the primary drivers of this change [1,2]. Among the most damaging invaders are social insects, especially ants, which often fundamentally alter the structure and dynamics of the invaded communities [3,4].

Interestingly, the introduction of the most damaging invasive ants is often accompanied by a shift to an extreme form of social organization, known as *unicoloniality* [5]. Unicolonial populations are comprised of nests that lack clear behavioral boundaries, contain many reproductive queens, and freely exchange individuals [6]. In its introduced range, the highly invasive Argentine ant (*Linepithema humile*) displays the most extreme form of unicoloniality known, as massive supercolonies can form across hundreds or thousands of kilometers, or even different continents [7,8,9,10]. Within these supercolonies, workers are genetically similar [11,12] and display virtually no aggression toward nestmates [7,9,13,14]. In the absence of the high costs associated with intraspecific aggression [15], more resources can be allocated to colony growth [13,16], giving introduced Argentine ants an advantage over native ant species [17,18]. Consequently, Argentine ants commonly cause a substantial reduction in the abundance of native ants [19,20,21,22] and other arthropods [4,23,24,25] which, in turn, has detrimental indirect effects on other species [26,27,28].

In their native South American range, Argentine ants do not possess the extreme unicolonial social organization that typifies introduced populations [7,12]. Instead, colonies range in size from a few meters to several hundred meters in diameter [7,12,29]. Moreover, native populations are genetically diverse [7,12] and highly differentiated [12,29], relative to introduced populations, and do not dominate native ecosystems [30].

The recognition cues used by ants to distinguish nestmates from foreign individuals are chemical pheromones, typically cuticular hydrocarbons (CHCs) that are expressed as a characteristic profile on the cuticle [31,32,33]. Members of a colony share a common chemical signature (“gestalt odor” [34]), created by the mixing of individual profiles through allogrooming, trophallaxis (i.e., mouth-to-mouth feeding) and physical contact [35]. It is believed that after worker ants emerge from pupation, they learn their colony’s recognition cues, thus, forming an internal template that is then compared to the profile of subsequently encountered conspecifics [36]. Individuals whose chemical signature deviates from the template are recognized as foreign and will usually be attacked. As for ants generally, Argentine ant colony recognition cues are CHCs [37,38] and the identity of some of the specific methyl-branched CHCs used for colony recognition have been identified using behavioral experiments with pure, synthetic CHCs [38]. Variation in the cuticular hydrocarbon pheromones that are used by Argentine ants to define colony membership [37] is correlated with genetic similarity and colony recognition behavior [39,40].

Although CHC variation appears to be genetically controlled in most social insects, there are also some examples of CHCs being acquired from food or nesting materials [41,42]. For example, an early paper on colony recognition in the leafcutter ant, *Acromyrmex octospinosus*, showed that shared diet and nesting materials reduced aggression among colonies [43]. Similarly, Richard et al. [44] showed in another leafcutter ant, *Acromyrmex subterraneus*, that dietary changes could affect colony recognition, perhaps through changes in the mutualistic fungus that is cultivated by the ants. Similar patterns have also been reported in various species of termites. When initially aggressive colonies of the Formosan subterranean termite, *Coptotermes formosanus*, are switched from different to similar diets, the levels of aggression they display toward each other are significantly reduced [45]. Interestingly, in the termite, *Reticulotermes speratus*, manipulations of the intestinal microbial community affect colony recognition behavior in predictable ways [46]. Thus, in the context of invasive Argentine ants, it is theoretically possible that environmental factors, such as changes in diet in introduced populations, could also contribute to the observed changes in Argentine ant social structure (the “dietary shift hypothesis”).

If prey-derived hydrocarbons are used as nestmate recognition cues, the manipulation of diet should produce predictable changes in behavior. Specifically, if ants from the same supercolonies are separated, then fed different insect diets, then they should begin to display aggression toward each other. Moreover, the opposite should also be observed: if behaviorally different colonies are fed the same diet, then they should begin to accept each other as colony mates.

The current evidence for the dietary shift hypothesis in Argentine ants is mixed. Suarez et al. [47] reared ants from several different, antagonistic supercolonies in California in the lab, on an identical diet, and tested the level of aggression among these lab colonies each month for a year. Despite this long period of rearing on the same diet, Suarez et al. did not see any evidence for behavioral homogenization coincident with the dietary homogenization. At the same time, ants from these lab colonies were tested monthly in behavioral assays, against newly collected ants from the source colonies in the field. No behavioral changes were seen in these tests either: the field and lab ants never started showing aggression toward each other, despite their different diets, except for one case of a colony boundary spatially shifting. These data suggest that Argentine ants do not use prey-derived hydrocarbons as recognition cues. However, a potential shortcoming of the aggression assays in this study is that a series of foreign ants were repeatedly introduced into the same lab colony. Previous studies have shown that such inter-colony encounters can affect the subsequent behaviors of Argentine ant workers [48].

More recently, Mothapo and Wossler [49] performed a laboratory experiment, in which introduced Argentine ants from two different supercolonies in South Africa were reared for five months on the same diet (pinhead crickets and sugar water). In this case, changes in aggression or recognition behavior were not observed: initially, aggressive supercolonies remained aggressive toward each other and ants in the lab colonies did not display increased aggression toward ants from the original sources in the field. Despite this behavioral stability, Mothapo and Wossler did observe changes in CHC profiles in the laboratory colonies, both gains of some CHCs from their insect diet and loss of other CHCs that were originally present in the field-collected ants.

In another laboratory experiment, Buczkowski et al. [50] reared initially aggressive Argentine ant colonies on two particular insect prey items (the roaches, *Blatella germanica* and *Supella longipalpa*) and an insect-free control diet, and monitored for reductions in aggression through time. *Supella longipalpa* was selected as a dietary treatment because previous studies had shown that it has an unusual effect on the CHC profiles and behavior of Argentine ants (see below). Buczkowski et al. found that, although CHC profiles of ants in these different dietary treatments changed through time, the behavioral consequences varied. Colonies that initially exhibited moderate levels of aggression showed reduced aggression after being reared on the same diet. However, colonies that displayed initially high levels of aggression showed only small (but significant) reductions in aggression after the feeding treatment; the post-treatment level of aggression was still sufficiently high to be physically injurious to the tested ants. Analysis of the CHC profiles showed that several hydrocarbons, specific to the insect prey items, were acquired by Argentine ants during the course of the experiment.

Other studies, frequently cited as evidence for the use of diet-derived cues, show the opposite pattern: initially non-aggressive Argentine ants from the same colony can develop aggression toward each other when fed one particular species of insect prey. This was first shown by Liang and Silverman [51], who recorded aggression between lab colonies and their sources in the field when the lab colonies were fed either *B. germanica* (German cockroaches) or *S. longipalpa* (brown-banded cockroaches) for 56 days. A subsequent study confirmed that feeding on *S. longipalpa* triggered immediate aggression among colony mates (within minutes), but this behavior was not observed for nine other species of insect prey, including *B. germanica* [52]. However, these results do not provide support for the idea that dietary shifts underlie the origin of supercolonies, since these treatments caused colony fragmentation rather than increased cooperation or reduced aggression.

In addition to the complications and mixed conclusions described above, previous studies of how diet-derived hydrocarbons may affect Argentine ant colony structure suffer from an additional shortcoming: since these are largely laboratory experiments, the dietary manipulations involve unnaturally high levels of insect prey supplementation. In the field, ant colonies are unlikely to experience a dietary shift as dramatic as that imposed in the laboratory, where a single insect species becomes nearly the sole food source.

In this study, we tested the dietary shift hypothesis for the origin of Argentine ant supercolonies by performing dietary manipulations of Argentine ant colonies, in both the field and the laboratory. To conclude that dietary components from prey are used for colony mate recognition cues requires: (1) that the recognition chemicals are transferred from prey to the ants and (2) that the presence of these new cues on the ant cuticle alters behavior. These behavioral changes can take two general forms: (1) reduced aggression between colonies of ants that shared the same diet (dietary homogenization leads to acceptance behavior) and (2) increased aggression between colony mates that have been fed different diets (dietary differentiation produces behavioral differentiation). Other outcomes, such as dietary homogenization leading to increased aggression, dietary differentiation leading to reduced aggression, or dietary differentiation leading to no behavioral change, would suggest that prey-derived odors are not generally used as colony mate recognition cues.

## 2. Materials and Methods

### 2.1. Dietary Manipulation in the Field and the Laboratory

#### 2.1.1. Field Experiment

The goal of this experiment was to test the effect of relatively minor dietary changes in a natural field setting (in contrast to the laboratory experiment below). We identified 32 *L. humile* nesting sites, all separated by at least 100 m, around the campus of the University of California-Irvine (Irvine, CA, USA). These ants were all behaviorally members of the large supercolony (CAL) that extends throughout most of the Argentine ant’s Californian range [7]. We randomly assigned each site to one of four dietary treatments: fruit flies (*Drosophila melanogaster*), cockroaches (*Blatella germanica*), crickets (*Acheta domesticus*), or cooked, scrambled chicken egg (“egg”, hereafter). We chose these prey species because they represent different insect orders, they are easily obtainable, and as common human commensals their abundance in Argentine ant diet is likely to naturally vary between nearby populations (particularly indoor vs. outdoor). Although, as noted above, previous studies have shown that feeding on *Supella longipalpa* can trigger intracolony aggression [51], we chose not to use this species because the behavioral response of Argentine ants appears to be unique to this type of insect prey. After an initial two-week monitoring period, each site was provided 0.1 g of its designated food item, placed on the ground, three times per week for four weeks. Although the food was not monitored to ensure consumption by Argentine ants, active foragers were present above ground and they likely recruited this food quickly. This mass translated to ~300–450 individual *Drosophila*, one large adult *Acheta*, or 4–5 adult *Blattella* per nest per week. Thus, these dietary subsidies represented a novel contribution to the normal dietary repertoire of the nests, but were not so large as to overwhelm the other natural food resources being retrieved by foragers. The nests selected for dietary supplementation were not large, dense aggregations, as can occur in some parts of the introduced range, but rather, were nesting sites generally located at the bases of trees or sidewalk edges with less suitable nesting habitat (e.g., open grass lawns, expanses of pavement) between them. Thus, the feeding treatments were likely distributed among the same limited population of hundreds to a couple thousand individual workers each week.

At the beginning of the experiment, we also collected large numbers of ants from two locations for use as stock colonies. One of these sites (William R. Mason Regional Park, Irvine, CA, USA) also belongs to the CAL supercolony, but is 1.5 km from the nearest treatment site. The other site (Lake Skinner County Park, Winchester, CA, USA) belongs to a different supercolony (LS) located ~75 km from the field treatment sites [14]. These stock colonies were maintained in the lab on a diet of sugar water, egg, protein solution (modified LB broth), and crickets (*Acheta domesticus*). Ants from the experimental treatments were tested in behavioral assays against ants from each of these stock colonies at five time points during the experiment (“Behavioral assays”, below).

#### 2.1.2. Laboratory Experiment

We collected the ants from Mason Park, Irvine (CAL) and divided them into 32 separate treatment nests, each containing approximately 1000 workers and two queens. Ants in these colonies were reared in large plastic tubs without soil or other debris. Nesting tubes were constructed of test tubes with a small amount of water at the bottom, and a cotton ball pressed down into contact with the water. We also collected and maintained new stock colonies, as described above. Each replicate was randomly assigned to one of the four dietary treatments. The ants in each tub were fed the same diet as the stock colonies for two weeks, then 0.1 g of their assigned food item was added. The ants were fed their assigned diet three times per week for four weeks, and then returned to the standard diet for two weeks.

### 2.2. Behavioral Assays

To test for an effect of diet on aggression we performed behavioral assays that paired ants from experimental treatments *versus* ants from the stock colonies. We quantified the level of aggression between workers using a standard behavioral assay [30].

For each assay, we placed two arbitrarily chosen workers in a Petri dish (3.5 cm diameter) that was lined with Fluon on the walls. We scored behavioral interactions for a five-minute period using the following categories: touch = 1 (physical contact); avoid = 2 (mandible flaring and contact that results in one or both of the workers to recoil and run in opposite direction); aggression = 3 (biting and pulling legs or antenna); fight = 4 (prolonged aggression >5 s often resulting in death or dismemberment). At the end of the five minutes, we recorded the maximum score attained. We used the mean value for a set of five trials for each of the colony pairings for the subsequent analysis.

For both the field and the laboratory experiments, we performed behavioral assays before starting the treatment (week two), during the treatment (weeks four, six, and eight), and after the treatment was discontinued (week ten). We compared aggression before, during, and after the treatment using Mann–Whitney U-tests.

### 2.3. Cuticular Hydrocarbon Analysis

We quantified the cuticular hydrocarbon (CHCs) profiles of Argentine ants before and during feeding treatments in both the field and lab for the *B. germanica* and *A. domesticus* treatments. We did not analyze CHCs of ants in the egg and *D. melanogaster* treatment because preliminary experiments showed essentially no shared CHCs after feeding. For ants in the *B. germanica* and *A. domesticus* treatments, we collected 10 workers from each dietary treatment at the beginning of the experiment (week two) and during the treatment period (week six) for analysis. We examined the CHC profiles of 24 individual workers from each sampled nest. We extracted CHCs by soaking an individual ant in 50 µL of hexane for 10 min. Two µL of the hexane extract was injected into a Finnigan Trace MS+ gas chromatograph/mass spectrometer equipped with a DB-5 capillary column (30 m × 0.32 mm × 0.25 µm, Agilent Technologies, Palo Alto, CA, USA). We used helium as carrier gas at 1 mL/min, the injector in splitless mode (1 min), and a temperature program of 2 min at 80 °C, to 270 °C at 20 °C/min, then to 310 °C at 3 °C/min. Injector temperature was set at 290 °C. Electron impact mass spectra were obtained with an ionization voltage of 70 eV and a source temperature of 250 °C. Thermo Finnigan’s Excalibur software (ver. 1.3) was used for data acquisition. Since the profile of Argentine ants consists largely of saturated hydrocarbons, we set up a method of Selected Ion Monitoring (SIM), narrowing the mass range scanned to the three ions 99, 113 and 127, which are highly specific for saturated hydrocarbons. Since each ion is scanned for a longer amount of time, this method is more sensitive than a full mass scan, and therefore yielded cleaner profiles. For analysis, we integrated chromatograms in the program ACD SpecManager (ver. 10.0, Advanced Chemistry Development, Toronto, ON, Canada) and standardized peak areas to percentages of the total area under the curve. For each supercolony, we also conducted GC-MS runs using full ion scans to obtain mass spectra for each peak. Compounds were then characterized using standard MS databases, diagnostic ions and Kovats indices [53].

We followed the same procedure to analyze CHC profiles of the different insect prey species. For the analysis of the prey items, we extracted hydrocarbons from approximately 50 *D. melanogaster*, one *B. germanica*, or one *A. domesticus* in 0.5 mL of hexane. Samples were analyzed using GC-MS. For each prey item, we ranked the CHC peaks by peak area and selected those that comprised 95% of the total peak area. We then scanned the worker profiles for these prey-specific peaks and, when present, calculated the peak area relative to the total area under the curve. We tested for the acquisition of prey hydrocarbons by Argentine ants that fed upon these insects using Mann–Whitney U-tests.

## 3. Results

### Dietary Manipulation in the Field and Laboratory

**Figure 1:**
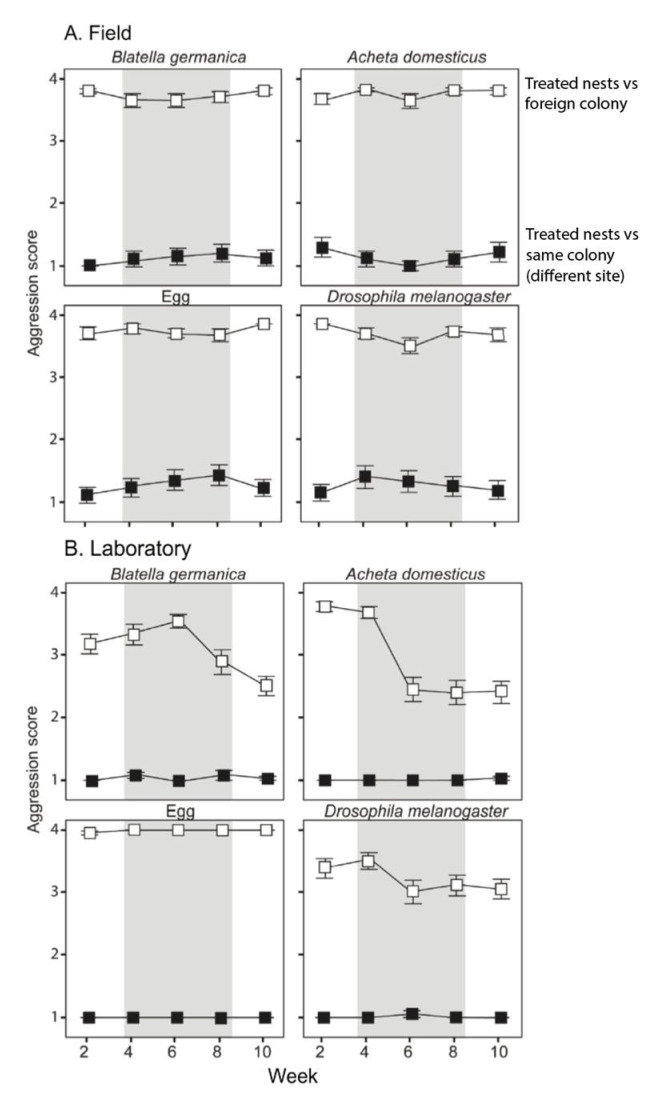
Levels of aggression through time in the dietary experiments (**A**) in the field and (**B**) in the laboratory. The heading of each sub−panel indicates the respective dietary treatment. Open squares show the level of aggression displayed by ants from treatment colonies (from the large Californian supercolony) toward ants from a foreign supercolony (Lake Skinner); Filled symbols show the level of aggression displayed by ants from treatment colonies toward ants from a different site (Mason Regional Park) that belongs to the same supercolony. Shaded portions indicate the time period during which the respective dietary items were provided.

In the field, we found that none of the four dietary treatments resulted in aggression between the treated nests and the laboratory stock from the large CAL supercolony, even after 10 weeks (Figure 1A, filled squares; Table 1, “Same supercolony”). Similarly, we did not see a reduction in aggression toward the laboratory stock of the LS colony in any of the four dietary treatments, across the entire course of the experiment (Figure 1A, open squares; Table 1, “Different supercolony”). Thus, in the field setting, similar diets did not make Argentine ants more amicable toward each other and different diets did not make them more aggressive toward each other.

In the laboratory feeding experiment, again, we found that none of the four dietary treatments resulted in aggression between the treated nests and the large CAL supercolony (Figure 1B, filled squares; Table 2, “Same supercolony”). However, in one of the four dietary treatments (cricket), we observed reduced aggression between the treatment and the ants from a different supercolony (Figure 1B, “A*cheta domesticus*”, open squares; *p* = 0.001; Table 2, “Different supercolony”). We did not observe a significant reduction in aggression toward non-colony members in any of the other three dietary treatments (Figure 1B, open squares; Table 2, “Different supercolony”). Thus, in the laboratory setting, different diets did not change the behavior of initially amicable ants toward each other, but similar diets made initially aggressive Argentine ants more amicable toward each other in one of the three dietary treatments.

Interestingly, the ants in the cricket treatment of the lab experiment were fed the same prey type as the large stock colonies had been fed, suggesting that increased dietary similarity may have decreased aggression between these supercolonies. However, even though the post-feeding level of aggression was significantly lower than the initial levels of aggression, the behaviors displayed between cricket-fed treatment ants and cricket-fed ants from the foreign supercolony were often injurious or fatal, and included lunging, biting, removal of legs and antennae, and use of chemical defenses.

**Figure 2:**
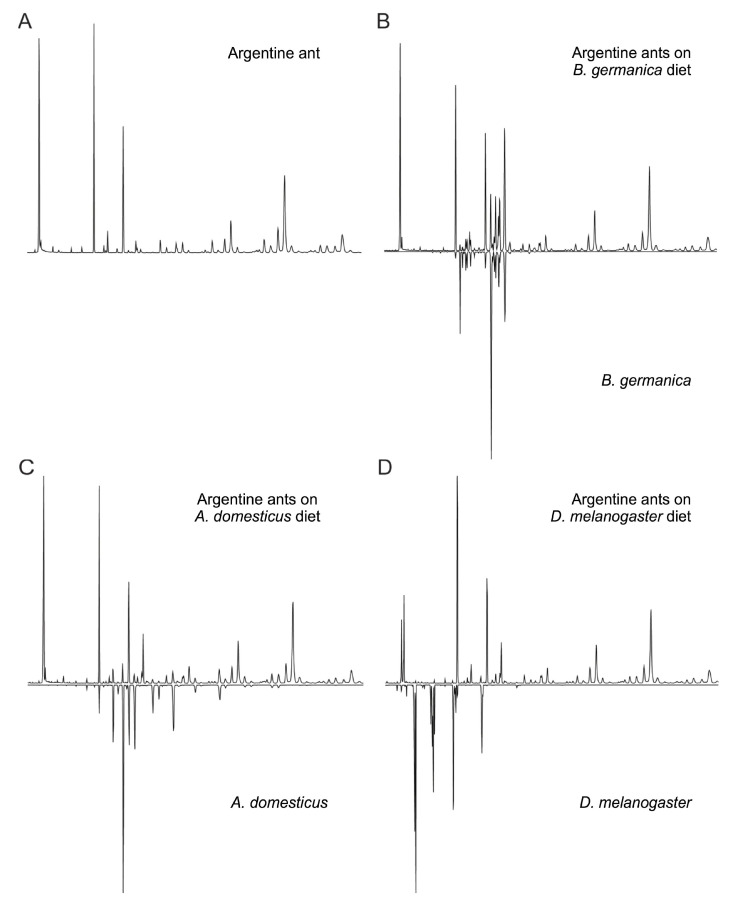
Mirror diagrams of cuticular hydrocarbon profiles from the dietary experiment. Upper profiles in each panel show cuticular hydrocarbon profiles of Argentine ants fed each type of insect diet; lower profiles are of the insect prey items themselves. (**A**) Unmanipulated Argentine ants, (**B**) *Blatella germanica*-fed Argentine ants (**top**) and *B. germanica* (**bottom**), (**C**) *Acheta domesticus*-fed Argentine ants (**top**) and *A. domesticus* (**bottom**), (**D**) *Drosophila melanogaster*-fed Argentine ants (**top**) and *D. melanogaster* (**bottom**).

To determine if Argentine ants acquired hydrocarbons from insect prey, we used gas chromatography to examine hydrocarbon profiles of two types of insect prey (*B. germanica* and *A. domesticus*) and compared these profiles to those of ants who fed on the respective prey species (as well as field-collected control ants). We found that prey hydrocarbons were not transferred to ants in the field experiment (“Field” in Table 3 and Table 4), but in the more extreme laboratory treatments, hydrocarbons from roaches and crickets did appear to transfer to the ants, changing their CHC profiles (Table 3 and Table 4, “Laboratory”; Figure 2B,C). Overall, the profiles of workers in the laboratory roach treatment showed a significant increase in 11 of the 12 most abundant roach cuticular hydrocarbons (Figure 2B, Table 3), and ants in the cricket treatment showed a significant increase in 4 of the 10 most abundant cricket hydrocarbons (Figure 2C, Table 4). No CHC transfer was seen in the *Drosophila* dietary treatment (Figure 2D).

## 4. Discussion

The ability of individuals to distinguish between group members and non-members is a defining feature of sociality [54]. Although chemical odors, particularly cuticular hydrocarbons (CHCs), are the most common and widespread indicators of colony membership in social insects, there are several potential origins of these cues.

In one straightforward model, insects produce an individual chemical signature that reflects their own genetic identity. When social insect colonies are comprised of kin, colony members will be chemically similar to each other, as a consequence of their heightened relatedness. In addition, individual differences can be homogenized across the colony membership through allogrooming, trophallaxis, and physical contact, producing some degree of chemical sharing.

Alternatively, when the nesting site, territory, and/or diet is shared by the members of a colony, and differs from that of other colonies, then odor cues derived from these environmental sources could be used as accurate indicators of colony membership. However, because such environmentally derived cues decouple colony membership from actual kinship, colony recognition systems that rely on environmental cues are at risk of invasion by non-kin (both conspecific and heterospecific) who happen to share the same environment. At the same time, if the environmental cues are not stable across space and time, environmentally based recognition systems can break down when the environment changes more quickly than the insects can adapt their recognition systems.

Our results, here, show that prey-derived hydrocarbons (one type of environmentally derived recognition cue) are unlikely to be used as colony recognition cues by Argentine ants in natural contexts. However, when extreme dietary shifts occur, as is often the case when colonies are reared in the lab, our data indicate that colony recognition behaviors can change. Thus, the results of our study reconcile the seemingly contradictory conclusions of several previous studies.

A series of previous studies have demonstrated that environmentally derived cues, in the form of CHCs from the brown-banded cockroach (*Supella longipalpa*), can affect Argentine ant colony recognition. In laboratory studies, it was observed that Argentine ants from the same nest display high levels of aggression toward each other, immediately after feeding on *S. longipalpa* [51,52]. Longer-term experiments revealed behavioral changes in laboratory colonies that experienced dietary manipulations [50]. However, several lines of evidence raise questions about whether these results support the conclusion that ant colonies use prey-derived CHCs for colony recognition.

First, as we show here, when dietary manipulation experiments are performed in a natural field setting, there are no observable changes to colony recognition behavior. Although several previous studies show behavioral and CHC profile changes in laboratory experiments, the magnitude of dietary change and the limited dietary breadth in such experiments is not representative of natural scenarios. Moreover, previous studies have shown that the transition of field-collected insects to a lab-rearing context can produce substantial changes to fundamental biological processes e.g., [55]. In natural habitats, Argentine ants (and nearly all other invasive ants) are renowned dietary generalists, feeding on a diverse array of different insect species, as well as carrion, floral nectar, homopteran exudate, fruit juices, and a wide array of anthropogenic food products and waste [4]. Thus, acquisition of new food items in the field likely occurs in the context of a diverse diet, thus, buffering the impact of any single new food on behavior or chemical profiles. The more limited amount of novel food provided in our field experiment was, therefore, not enough to overwhelm any colony recognition cues that may be derived from these other natural food sources. We argue that caution is warranted when extrapolating from the results of laboratory feeding experiments to conditions in the wild.

Second, it is hard to imagine how the intense intracolony aggression displayed by Argentine ants after feeding on *S. longipalpa* could be adaptive, particularly because it produces high levels of mortality within colonies. In fact, this behavior exemplifies the breakdown of colony recognition rather than a functional, adaptive recognition system. It appears that this aggressive response is simply a consequence of workers becoming contaminated with *S. longipalpa* CHCs after contact with prey, resulting in them being mistaken by nestmates as intruders.

Third, the behavioral responses to feeding on *S. longipalpa* appear to be unique to this prey species. Similar experiments with other types of insect prey did not provoke the same types of responses. In a previous experiment, Liang et al. [52] tested the behavior of Argentine ants after being fed a variety of different insect prey, including *Blattella germanica*, *Supella longipalpa*, *Musca domestica* (house flies), *Incisitermes minor* (western drywood termites), *Zootermopsis nevadensis* (Nevada dampwood termites), *Reticulitermes flavipes* (eastern subterranean termites), *Oncopeltus fasciatus* (large milkweed bugs), *Acheta domesticus*, and *Tenebrio molitor* (mealworm beetles). The intracolony aggression observed after feeding on *S. longipalpa* was not seen after Argentine ants fed on any of the other insect species. However, at present, only a handful of insect prey species have been tested for potential impacts on CHC profiles or colony recognition behavior, and it is possible that other species may produce the effects seen in response to *S. longipalpa* prey.

Fourth, colony boundaries between Argentine ant supercolonies are sharply delineated in space and characterized by high aggression, despite these ants sharing the same environment, microhabitats, and local diet. In fact, aggressive behavior at colony boundaries is significantly higher than between naïve ants from more distant locations [15] because previous aggressive encounters with non-colony mates cause workers to show more aggression in subsequent encounters [48].

Fifth, in the more natural setting of our field experiment, none of the dietary treatments caused a significant reduction in aggression toward ants from a foreign supercolony that was fed the same diet (Figure 1A, open squares; all *p* > 0.05). Thus, although the direction of the behavioral change seen in the cricket treatment in the laboratory experiment is consistent with the dietary shift hypothesis, the high levels of aggression, even after the behavioral shift, and the absence of this pattern in the field both suggest that a shift to new prey in the introduced range is unlikely to be responsible for the origin of widespread unicoloniality. Even if different supercolonies that consume the same prey are less aggressive toward each other (which the results of the field experiment suggest is not the case), they still attack each other, and would be unlikely to fuse together into a single cooperative supercolony.

Finally, even after extreme dietary homogenization in our laboratory feeding experiments, aggression was never eliminated between initially aggressive supercolonies. Although one dietary treatment produced significantly reduced aggression, the levels of aggression were still high (as discussed above). In this respect, our results match those previously reported by Mothapo and Wossler [49], in which a five-month feeding experiment on crickets and sugar water did not produce reduced aggression between different supercolonies (but did result in CHC changes). Thus, it is hard to imagine a natural scenario, in which dietary homogenization would produce widespread colony fusion, and hence, the origin of unicoloniality.

Although our results, and those in previous studies, shed light on the role of diet in colony recognition and social insect chemical ecology, many important questions remain unanswered. For example, it is clear that Argentine ants (and probably other social insects) can, under some circumstances, acquire CHCs from insect prey, but little is known about how this may vary across specific CHCs or CHC families. Similarly, when colony recognition is disrupted (as when Argentine ants feed on *S. longipalpa* roaches), what are the features of these CHCs that cause these behavioral effects, when others do not?

## Figures and Tables

**Table 1:** Mean level of aggression (±SD), before, during and after the dietary experiments in the field. Z scores and *p* values are from Mann-Whitney U-tests.

		Before		During		After		Before vs. During		During vs. After	
Opponent	Treatment	Mean ± SD	n		n		n	Z	*p*	Z	*p*-
Same supercolony	*A. domesticus*	1.43 ± 0.53	8	1.23 ± 0.45	7	1.34 ± 0.53	7	−0.853	0.393	−0.597	0.551
	egg	1.22 ± 0.31	8	1.55 ± 0.63	8	1.35 ± 0.40	8	−1.069	0.285	−0.541	0.588
	*D. melanogaster*	1.28 ± 0.44	8	1.37 ± 0.53	7	1.12 ± 0.30	7	−0.393	0.694	−0.07	0.944
	*B. germanica*	1.15 ± 0.23	8	1.33 ± 0.44	8	1.25 ± 0.30	8	−0.756	0.45	−0.171	0.864
Different supercolony	*A. domesticus*	3.83 ± 0.20	8	3.94 ± 0.15	7	3.94 ± 0.15	7	−1.326	0.185	0	1
	egg	3.85 ± 0.28	8	3.83 ± 0.27	8	4.00 ± 0.00	8	−0.387	0.669	−1.852	0.064
	*D. melanogaster*	4.00 ± 0.00	8	3.89 ± 0.23	8	3.83 ± 0.29	7	1.565	0.118	−0.161	0.876
	*B. germanica*	3.95 ± 0.09	8	3.85 ± 0.23	8	3.95 ± 0.14	8	−0.769	0.442	−1.105	0.269

**Table 2:** Mean level of aggression ± SD, before, during and after the dietary experiments in the laboratory. Z scores and *p* values are from Mann-Whitney U-tests. Bold indicates *p* < 0.05.

		Before		During		After		Before versus During		During versus After	
Opponent	Treatment	Mean ± SD	n		n		n	Z	*p*-	Z	*p*
Same supercolony	*A. domesticus*	1.00 ± 0.00	8	1.00 ± 0.00	8	1.25 ± 0.07	8	−1	0.317	−1	0.317
	egg	1.00 ± 0.00	8	1.00 ± 0.00	8	1.00 ± 0.00	8	0	1	0	1
	*D. melanogaster*	1.00 ± 0.00	8	1.00 ± 0.00	8	1.00 ± 0.00	8	0	1	0	1
	*B. germanica*	1.00 ± 0.00	8	1.08 ± 0.21	8	1.03 ± 0.07	8	0	1	−0.091	0.927
Different supercolony	*A. domesticus*	3.38 ± 0.36	8	3.10 ± 0.19	8	3.05 ± 0.26	8	−3.401	**0.001**	0	1
	egg	3.78 ± 0.20	8	2.40 ± 0.44	8	2.40 ± 0.37	8	1.464	0.143	0	1
	*D. melanogaster*	3.95 ± 0.09	8	4.00 ± 0.00	8	4.00 ± 0.00	8	−1.846	0.083	−0.113	0.91
	*B. germanica*	3.18 ± 0.40	8	2.88 ± 0.54	8	2.50 ± 0.32	8	−1.124	0.261	−1.807	0.071

**Table 3:** Mean ± SD relative proportion of *B. germanica* cuticular hydrocarbon peak areas in *L. humile* profiles before and during the dietary treatment. Z scores and *p* values are from Mann-Whitney U-tests. Bold indicates *p* < 0.05.

	Laboratory				Field			
Cuticular	Before	During			Before	During		
Hydrocarbon	Mean ± SD	Mean ± SD	*Z-*Value	*p*-Value	Mean ± SD	Mean ± SD	*Z-*Value	*p*-Value
1	0.000 ± 0.000	0.005 ± 0.005	−3.59	**<0.001**	0.000 ± 0.000	0.000 ± 0.000	0	1
2	0.000 ± 0.000	0.003 ± 0.002	−3.59	**<0.001**	0.002 ± 0.002	0.002 ± 0.001	0	1
3	0.005 ± 0.001	0.009 ± 0.005	−2.406	**0.016**	0.000 ± 0.000	0.000 ± 0.000	0	1
4	0.000 ± 0.000	0.007 ± 0.005	−3.59	**<0.001**	0.000 ± 0.000	0.000 ± 0.000	0	1
5	0.000 ± 0.000	0.006 ± 0.004	−3.59	**<0.001**	0.000 ± 0.000	0.000 ± 0.000	0	1
6	0.106 ± 0.023	0.103 ± 0.048	−0.21	0.834	0.055 ± 0.024	0.053 ± 0.026	−0.105	0.916
7	0.000 ± 0.000	0.046 ± 0.029	−3.59	**<0.001**	0.000 ± 0.000	0.000 ± 0.000	0	1
9	0.000 ± 0.000	0.018 ± 0.018	−3.59	**<0.001**	0.000 ± 0.000	0.000 ± 0.000	−0.316	0.752
9	0.000 ± 0.000	0.037 ± 0.021	−3.59	**<0.001**	0.000 ± 0.000	0.000 ± 0.000	0	1
10	0.010 ± 0.004	0.058 ± 0.044	−3.361	**0.001**	0.001 ± 0.002	0.001 ± 0.002	−0.231	0.817
11	0.000 ± 0.000	0.041 ± 0.020	−3.59	**<0.001**	0.000 ± 0.000	0.000 ± 0.000	0	1
12.	0.000 ± 0.000	0.106 ± 0.061	−3.59	**<0.001**	0.000 ± 0.000	0.000 ± 0.000	0	1

**Table 4:** Mean ± SD relative proportion of *A. domesticus* cuticular hydrocarbons peak areas in *L. humile* profiles before and during the dietary treatment. Z scores and *p* values are from Mann-Whitney U-tests. Bold indicates *p* < 0.05.

	Laboratory				Field			
Cuticular	Before	During			Before	During		
Hydrocarbon	Mean ± SD	Mean ± SD	Z-Value	*p*-Value	Mean ± SD	Mean ± SD	Z-Value	*p*-Value
1	0.273 ± 0.148	0.192 ± 0.087	−0.926	0.355	0.130 ± 0.175	0.114 ± 0.087	−0.575	0.565
2	0.030 ± 0.016	0.019 ± 0.010	−1.389	0.165	0.016 ± 0.028	0.014 ± 0.007	−1.023	0.306
3	0.015 ± 0.041	0.000 ± 0.000	−0.935	0.35	0.000 ± 0.000	0.000 ± 0.000	0	1
4	0.178 ± 0.151	0.120 ± 0.041	−0.694	0.487	0.016 ± 0.034	0.000 ± 0.000	−1.468	0.142
5	0.000 ± 0.000	0.007 ± 0.013	−1.565	0.118	0.000 ± 0.000	0.000 ± 0.000	0	1
6	0.000 ± 0.000	0.004 ± 0.008	−1.985	**0.047**	0.000 ± 0.000	0.000 ± 0.000	0	1
7	0.000 ± 0.000	0.001 ± 0.002	−1.985	**0.047**	0.000 ± 0.000	0.000 ± 0.000	0	1
8	0.000 ± 0.000	0.000 ± 0.000	0	1	0.000 ± 0.000	0.000 ± 0.000	0	1
9	0.001 ± 0.002	0.008 ± 0.004	−3.227	**0.001**	0.000 ± 0.001	0.000 ± 0.000	−1	0.317
10	0.000 ± 0.000	0.032 ± 0.018	−3.133	**0.002**	0.000 ± 0.000	0.000 ± 0.000	0	1

## Data Availability

Raw behavioral data are available at Dryad (www.datadryad.org), https://doi.org/10.6078/D1070P (accessed on 1 February 2022).
